# The Consumption of Caffeine-Containing Products to Enhance Sports Performance: An Application of an Extended Model of the Theory of Planned Behavior

**DOI:** 10.3390/nu13020344

**Published:** 2021-01-24

**Authors:** Antonella Samoggia, Tommaso Rezzaghi

**Affiliations:** Department of Agro-Food Sciences and Technologies, Alma Mater Studiorum University of Bologna, Viale Fanin 50, 40127 Bologna, Italy; tommaso.rezzaghi2@unibo.it

**Keywords:** consumer, behavior, perception, health, caffeine, theory of planned behavior, sport, performance

## Abstract

Caffeine is the most-used psychoactive substance in the world. About 80% of the world’s population consumes caffeine every day, including athletes and lifestyle users. Thus, it is important to understand the consumer drivers of caffeine-containing beverages and food. This research study aims to explore consumers’ behaviors, perceptions, attitudes, and drivers towards caffeine-containing products to enhance sports performance. The research applies the Theory of Planned Behavior (TPB) in order to understand consumers’ behavior, extended with utilitarian aspects for a comprehensive understanding of consumers’ behavior and attitudes. We interviewed consumers with the support of Qualtrics online software. The data were then processed with SPSS (statistical analysis software). The data elaboration includes a multivariate linear regression model to analyze the consumers’ intention to consume caffeine to enhance the sports performance, and to explore consumers’ preference of marketing leverages for this product category. The results contribute to an understanding of consumers’ consumption and purchasing behavior towards caffeine, and support the validity of the extended TPB to develop a more comprehensive picture of consumer behavior. Consumers have a positive attitude towards caffeine-containing products to enhance sports performance. The main consumer behavior drivers are subjective norms and utilitarian aspects. The present research results may support companies in the development of caffeine-containing products to enhance sports performance.

## 1. Introduction

Caffeine is the most-used psychoactive substance in the world. About 80% of the world population consumes caffeine every day, with a daily intake equal to 200 mg, equivalent to around three espresso cups per day [1,2,3]. It can be found naturally in the seeds, fruits and leaves of more than 60 species of plants, including the coffee plant (*Coffea canephora* and *Coffea arabica*), tea plant (*Camellia sinensis*), cocoa plant (*Theobroma cacao*), yerba mate (*Ilex paraguariensis*) and kola plant (*Cola nitida*), as well as being produced artificially [4]. In chemical terms, caffeine is a pure alkaloid, which means that it is a basic and organic plant-derived substance, with the chemical name of 1,3,7-trimethylxanthine. It is an odorless white powder that is soluble in both water and lipids, characterized by a bitter taste. Although it has no nutritive value, it has become an important additive in a range of food and drinks, such as coffee, tea, chocolate, mate, Guarana and cola nut. Caffeine is also present in energy drinks and sport food, where it is added. The actual content of caffeine of these products is variable, and depends on the quality and type of processing of the raw materials [5].

Caffeine is socially recognized as a ‘stimulant’, and it is mainly used to improve both cognitive functions and physical performance [6,7]. Caffeine is the most popular of all sporting ergogenic aids, and it is consumed by 75% of athletes before or during the competition [8,9]. The most well-established mechanism by which caffeine exerts its effects on sports performance is its role as a competitive adenosine receptor antagonist [10]. Due to these characteristics, caffeine has been considered, for a long period, a doping substance.

Caffeine-containing products are consumed at global level, are common among different consumers’ age groups, and are consumed for a number of reasons, from socialization to mental and physical alertness [7,11]. Recent research studies have pointed out the positive effect that caffeine has on sport performance [12,13]. Whereas there is increasing research on consumers’ perception towards caffeine-containing products, such as coffee, there is limited knowledge and understanding of consumers’ behavior and attitude towards caffeine-containing products to enhance sport performance. Thus, the objectives of the current research are to analyze: (i) consumers’ behaviors, perceptions, attitudes, and drivers towards caffeine-containing products to enhance sport performance; (ii) caffeine consumption and purchasing motives; and (iii) the market channels and formats of caffeine-containing products to enhance sport performance.

The structure of the paper is as follows. Section 2 provides a literature review of studies providing evidence on caffeine and sport performance, sports nutrition market trends, research on caffeine consumption and purchasing motives, and consumers’ behavior towards caffeine-containing products. Section 3 describes the data gathering and elaboration, and the data sample. The results are presented in Section 4. This section first discusses the results regarding consumers’ consumption habits and motives with regard to caffeine, and consumers’ intention to consume caffeine-containing products to enhance sport performance, followed by insights on sales strategies for caffeine-containing products to enhance sport performance. Finally, the paper provides a discussion on the available literature on consumers’ perceptions of caffeine’s health effects. The conclusive comments put the topic into the broader context of consumers’ increasing interest in caffeine-containing products and dietary behavior, and reflects on the marketing possibilities for caffeine-containing products.

## 2. Literature Review

### 2.1. Caffeine in Sports

#### 2.1.1. Caffeine Consumption and Its Effects on Sport Performance

There is evidence that caffeine enhances sports performance [14]. In particular, the EFSA (European Food Safety Authority) confirmed that there is a cause and effect relationship between caffeine intake and increased endurance performance, endurance capacity, and a reduction in perceived exertion [15]. The effectiveness of caffeine on sports performance depends on three main factors: the dose and timing of administration; the type of sports; and the individual’s response to caffeine [16]. First, a common question for sport people interested in caffeine supplementation is “What dose of caffeine should I use?”. The studies addressing this issue achieved controversial results. There is no linear dose–response relationship between the caffeine dose and the magnitude of its ergogenic effects [17]. The International Society of Sports Nutrition’s position on caffeine is that caffeine is effective at enhancing sports performance at moderate dose (3–6 mg/kg of body weight), with no added benefits on the performance at doses higher than 9 mg/kg of body weight [18]. The effect of a moderate dose of caffeine has pushed the research to investigate the effectiveness of lower doses (<3 mg/kg of body weight) [12]. The review carried out by Spriet on the relationship between exercise performance and low doses of caffeine [19], and the meta-analysis carried out by Souza and colleagues on the ergogenic effects of caffeine-containing energy drinks, concluded that these lower doses of caffeine enhance sports performance [20]. The extent to which low doses of caffeine are ergogenic as a moderate dose is not clear. More studies should be carried out to provide a consistent picture [12]. The achievement of the effectiveness of the caffeine dose should take into account the different times of administration, such as before the event, throughout the event, and toward the end of the event [16]. Second, caffeine seems to be effective in enhancing performance in most sports, with the greatest effects being found in sports that involve intense fatigue during or toward the end of the event. However, caffeine does not seem to be beneficial for those events characterized by very high intensity, or a power output lasting seconds, such as sprints or lifts [16]. Third, people vary in their responses to caffeine ingestion, ranging from positive, to neutral and negative effects on performance. This variability depends on the genotype, training status, habitual use of caffeine, gender, caffeine source, and age [12]. Past findings support that the responses to caffeine vary on an individual basis; as such, it is difficult to make a clear recommendation in terms of dosage.

#### 2.1.2. Caffeine Permissibility and Doping

In the sports field, an ergogenic aid is defined as a technique or a substance that allows athletes to enhance their performance [21]. Caffeine is an example of an ergogenic aid; more specifically, it is a stimulant. For this reason, antidoping organizations have always monitored and evaluated it. From 1984 to 2004, caffeine was included in the prohibited list. In particular, from 1985, there was a set threshold for the concentration of caffeine in the urine of athletes equal to 12 μg/mL. This cut-off value was chosen to exclude normal caffeine consumption, and to differentiate it from what was considered an inadequate use of caffeine for the purpose of enhancing sports performance [16]. The most important change in the permissibility of caffeine in sport was approved by the World Anti-Doping Agency (WADA). WADA is an international independent agency including the sports associations and governments of the world, which was established in 1999. Its activities include scientific research, education, the development of anti-doping capacities, and the monitoring of the World Anti-Doping Code (the document harmonizing policies in all sports and countries). The World Anti-Doping Code, developed by WADA, came into effect on 1 January 2004. It was prepared appositely for the Athens Olympic Games, and remains the current standard. The World Anti-Doping Code is the core document that comprises anti-doping policies, rules and regulations within sports organizations and among public authorities around the world. It sets a prohibited list which contains prohibited substances and methods in and out of the competition. A substance or a method is included in the prohibited list if it satisfies any two of the following criteria: it has the potential to enhance or enhances sports performance; it represents an actual or potential health risk to the athlete; or it violates the spirit of sports. The prohibited list is reviewed every year, and it comes into effect on 1 January of each year [22]. Caffeine was removed from the prohibited list in 2004, so currently its use in sports is not prohibited. However, caffeine was included in last year’s WADA monitoring programs. The WADA monitoring program involves substances that are currently off of the prohibited list, but which WADA wants to monitor and check in order to detect any pattern of misuse in sports. Caffeine was present even in the 2020 WADA monitoring program [22].

### 2.2. Sports Nutrition Market

Sports nutrition is a key field of modern sports medicine, which helps athletes to maintain their bodies in good condition before, during, and after exercise in order to achieve the best possible performance [23]. It is important to note that sports nutrition is a well-known issue. Since the Ancient Olympics of 500 BC, athletes have recognized their enhanced requirement for nutrients by eating massive quantities of meat, bread, dried fruits and honey, along with various fungi and herbs in an attempt to support athletic performance [24]. The modern sports nutrition market is agreed to have been established in 1940 with the advent of Weider Global Nutrition [24], a nutrition company based in Phoenix, Arizona with over 70 years of expertise [25].

Nowadays, global trends and consumer concerns are defining changes in nutrition, and thereby influencing the sports nutrition category as well. People are starting to pay more attention to healthy nutrition and physical exercise to delay aging, and to prevent chronic diseases and premature mortality [26]. The consequence is that more people are adopting an active lifestyle and recognizing the benefits of sports nutrition products to complement their work-out session [27]. The so-called core users—that is, professional athletes and bodybuilders—are not the only ones using sports nutrition products. The growing health consciousness and desires for rapid performance achievements have helped these products to become more appealing to mainstream consumers over the last decade [28]. This ‘mainstreaming phenomenon’ has driven the sports nutrition market to a significant sales growth between 2004 and 2018, equal to +190% [29]. In 2019, the sports nutrition market was worth $17 billion, and it is expected to reach $21 billion by 2023, with a compound annual growth rate (CAGR) of 7.9 % [30].

The increasing popularity of sports nutrition products has driven the European Parliament and the European Council to mandate a report to the European Commission to better understand the market of food intended for sportspeople (FISP), and the need for requirements to regulate the market [31]. FISP has been categorized into three main categories: sports-drinks, protein-based products, and energy- and performance-boosting products. The value of each category on the market, as registered in the 2014, was 1858 Million Euros for sports drinks, 801 Million Euros for protein-based products, and 406 Million Euros for energy- and performance-boosting products [31]. Caffeine-containing products to enhance sports performance are included in the ‘performance boosting products’ category. This means that, beyond caffeine-containing common food and drinks, consumers can ingest caffeine through many other food sources, to which it is artificially added [16].

### 2.3. Consumers’ Behavior Towards Caffeine-Containing Products

Energy products containing caffeine are increasingly demanded by different population groups, including athletes, students and workers. They are commonly used to enhance people’s energy, concentration, and athletic performance. They can be easily purchased on the internet, in supermarkets, sports nutrition shops, and herbalist shops [32]. However, there are few studies investigating the consumer behaviors and preferences towards caffeine-containing products to enhance sports performance.

The aim of this research is to contribute towards the filling of this gap. In particular, it aims to better understand consumer behaviors regarding the caffeine-containing products used to enhance sports performance—such as the energy bars, gels, soluble powder, energy shots, pills, and chewing gum generally used by sportspeople—and more traditional food and drinks containing caffeine—such as coffee, tea, chocolate, soft drinks, and energy drinks—the consumption of which is more frequently driven by hedonistic values. Energy drinks are a relatively new category in the wider soft drinks market, and some types of energy drinks can be considered functional beverages [33,34]. They are non-alcoholic beverages containing high levels of caffeine (>150 mg/l), and are explicitly marketed as a way to relieve fatigue and improve mental awareness [35]. A recent study conducted by Verster and Koenig considers coffee, tea, and soft drinks to be the most important daily caffeine sources [36]. Coffee, in particular, is the most frequently-consumed caffeine-containing beverage, and represents the main daily source of caffeine in several countries [7]. Energy drinks have only a minor contribution to the total caffeine intake among all age groups (children, adolescents and adults) [36]. A study conducted on adolescent and young adult perceptions of caffeinated energy drinks found out that the youngest group of participants (16–21 years of age) showed a more frequent consumption of energy drinks in comparison to those in the oldest age group (29–35 years of age) [37]. Similar results were obtained by Drewnowsky and Rehm’s study, which tried to identify the sources of caffeine in the diets of US children and adults. The largest contributors to dietary caffeine were coffee (64%), tea (18%), and caffeinated sodas (15%). Energy drinks and foods containing caffeine accounted, respectively, for 2% and 1.5% of the total dietary caffeine [38].

Thus, the issue is understanding the consumer drivers of caffeine-containing beverages and food. A recent piece of research conducted on the motives for caffeine consumption showed that they can be grouped into six main motives. The taste of caffeine-containing products was the first motive of consumption, followed by habit, alertness, social, mood, and symptom-management motives [11]. Alertness, which is the main motive of consumption linked to physical performance and sports, was the third main motive pushing people to consume caffeine-containing products. This confirms the consumer belief that caffeine can help them to achieve better physical performance, and to attain better results in sports [7,39].

## 3. Theoretical Framework

The theoretical framework of the current study is based on the theory of planned behavior (TPB), developed by Ajzen [40]. The goal of the TPB is to predict and understand consumer consumption behavior. The TPB is an extension of the theory of reasoned action (TRA) [41,42], and its central factor is the individual’s intention to perform a specific behavior. Intentions are expected to capture the motivational factors that influence a behavior. They are indications of how hard people are willing to try, and of how much of an effort they are planning to exert, in order to perform the behavior in question. The assumptions are that the stronger the intention to engage in a behavior, the more likely its performance should be [40].

The TPB assumes that the immediate determinant of a behavior is the intention to perform the behavior, based on an intention–behavior correlation. In order to increase the intention’s prediction accuracy, the intention must be determined at all levels. According to the theory of reasoned action (TRA) elaborated by Ajzen and Fishbein, the intention is a direct function of two independent determinants: the attitude toward a behavior, and the perceived subjective norm regarding a behavior [42]. Subsequent research found out that the TRA was not as effective in the prediction of a behavior that was not under complete control. For this reason, Ajzen proposed the addition of perceived behavioral control as a fourth construct.

Therefore, the more advanced model assumed that the determining factors of behavioral intention are mainly three: attitude, subjective norms, and perceived behavioral control. First, the attitude (ATT) toward the behavior refers to the degree to which a person has a favorable or an unfavorable evaluation or appraisal of the behavior in question [40]. The intention to perform a behavior is affected by the attitude toward the behavior. The attitude of a person will vary depending on his evaluation of the probability to obtain a positive or a negative outcome. If the person expects a positive outcome, he is more likely to perform the behavior [43]. Second, subjective norms (SN) are a function of an individual’s normative beliefs, which are linked to the perceptions of the social pressures that push the person to perform the behavior [40]. Normative beliefs depend on the presence of people who are important to the individual, and on the motivation to comply with the wishes of these people. If people who are important to an individual want them to perform a specific behavior, and the individual intends to conform to their want, then it is expected that the individual will perform the behavior in question [43]. Third, the perceived behavioral control (PBC) is a function of an individual’s control beliefs. These control beliefs consist of the presence or absence of the individual’s resources and opportunities. These can be based partly on the past experience of the behavior, influenced by second-hand information, and on other factors that increase or reduce the perceived difficulty of the performance of the behavior in question [43].

**Figure 1:**
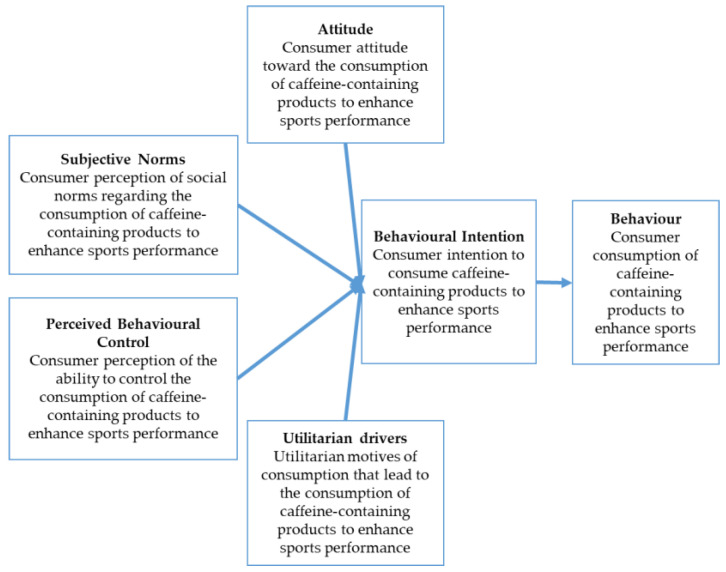
Extended model of the Theory of Planned Behavior.

The current study focuses on consumption behavior regarding caffeine-containing food products to enhance sports performance. The intention to consume caffeine-containing food products to enhance sports performance is the dependent variable. The determinants of consumers’ intentions are: subjective norms, attitude, and the perceived behavioral control towards caffeine and caffeine-containing products to enhance sports performance. The TPB framework applied in the present research was extended by the addition of a fourth determinant of the caffeine-containing product consumption intention. This added construct is based on utilitarian theories. The aim was to explore a possible correlation between the behavior-intention and the utilitarian motives, and drivers of caffeine consumption to improve sports performance (UD). Utilitarian drivers focus on the product’s capability to satisfy helpful, necessary and practical needs. The intention to consume and purchase products is influenced by the caffeine-containing product’s ease of use, accessibility, satisfaction, and practical use [44,45,46]. This set of drivers, together with the three TPB construct determinants, influence the dependent variable, which is the behavioral intention to consume caffeine-containing products to improve sports performance (Figure 1).

As a synthesis, the adopted formula representing the TPB is the one below:

INT = w1ATT + w2SN + w3PBC + w4UD

where INT = behavior intention; ATT = attitude toward a behavior; SN = the subjective norm; UD = utilitarian drivers; and w1, w2 and w3 are empirically-determined weights [40].

## 4. Materials and Methods

### 4.1. Data Gathering

The data was collected using a questionnaire with closed-ended questions, which was filled in by a convenience sample of consumers. The questionnaire included seventeen questions containing various items. The possible answers were single answer questions, multiple answer questions, a bipolar question, and questions which provided a 7-point Likert scale to express the level of agreement (1: ‘Totally disagree’ to 7: ‘Totally agree’, with scale end values anchored to the interpretations). The questionnaire was tested in trial online interviews, and the items that were identified as unclear or unimportant were revised. Social networks were employed in order to promote the survey. The data collection phase was carried out from July to September 2020. The time that was necessary to carry out each interview was around seven minutes. No reward or token was awarded. The data were collected with the support of the Qualtrics survey program, a software specialized in online surveying.

The questionnaire included four sections (Table 1). The first section explored consumers’ caffeine consumption habits and physical activity. The aim was to collect information about how frequently and what types of food products containing caffeine are consumed by the interviewees, and the main reasons why they consume them. Moreover, it collected information on the frequency of consumers’ physical activity, and what type of sports and physical activity they practice.

The second section explored consumers’ behavior, and perception of caffeine consumption for sport performance. It applied the extended TPB [40]. It aimed to understand consumers’ behavior regarding the consumption of caffeine-containing products to enhance sport performance exploring the intention (INT), attitude (ATT), perception regarding subjective norms (SN), perceived behavioral control (PBC), and utilitarian motives of consumption (UD). The consumer intention to ingest caffeine-containing products to enhance sports performance (INT) was measured by five items. People’s attitude towards the consumption of caffeine-containing products to enhance sports performance (ATT) was measured by four items. The perceived behavioral control regarding the consumption of caffeine-containing products to enhance sports performance (PBC) was measured by five items. The subjective norms regarding the consumption of caffeine-containing products to enhance sports performance (SN) was measured by five items. The utilitarian drivers related to the consumption of caffeine-containing products to enhance sports performance (UD) were measured by four items.

The third section collected marketing information about the investigated product. In particular, the survey gathered marketing information related to consumers’ favorite type of caffeine-containing product, favorite sport performance benefits associated with caffeine, favorite market channels, and favorite advisors on caffeine ingestion aimed at sport performance.

The last section aimed to gather information on the interviewees’ sociodemographic information, that is, age, gender, and level of education. The questionnaire items were developed based on a tailored literature review (Table 1).

### 4.2. Data Elaboration

The data elaboration followed different phases. First, a descriptive statistics analysis was performed in order to characterize the sample of interviewees according to their gender, age, level of education and frequency and type of physical activity. Second, the data elaboration calculated the mean values and standard deviations of the constructs and related items, according to the level of relevance expressed by the people (1–7 points Likert scale). Cronbach’s alpha values were calculated in order to test the consistency and the reliability of the constructs. Third, the construct values were used in the multivariate linear regression model, which was carried out in order to explore the relationships between people’s intention to consume caffeine-containing products to enhance sports performance (INT) and its drivers (ATT, SN, PBC and UD). The research checked for possible multicollinearity among the construct variables, in order to verify the possibility that one variable is a linear function of the other. The multicollinearity was tested through tolerance and variable inflation factors (VIFs). The reliability of the model was verified by the R^2^ test and the F-test. The results confirmed the validity of the model. Finally, in order to elaborate the marketing information and preferences data about caffeine-containing products for sport performance, the researchers performed a multiple response analysis. The data elaboration was carried out with the support of SPSS (statistical analysis tool, by IBM Corp., version 23).

### 4.3. Sample

The data cleaning led to the definition of a set of questionnaires for the data elaboration. The initial sample included 369 people. After a data validity check which aimed to exclude incomplete questionnaires, the final sample included 273 questionnaires used for data elaboration. The final sample included a good balance of men and women. The average age of the interviewees was around 29 years old, and more than half of the respondents had a university degree. The vast majority of the respondents practiced a sport activity (Table 2).

## 5. Results

### 5.1. Consumers’ Caffeine Consumption Habits and Motives

The results showed that coffee is the caffeine-containing product that was consumed the most by the sample (Table 3). More than 75% of interviewees drank coffee at least once a day, and only 10.5% of the sample never drank it. Tea was consumed less regularly than coffee. Less than 20% of the sample consumed tea at least once a day, and 26% of the sample never drank it. Similarly to tea, 20% of the sample consumed chocolate at least once a day, and 25.8% of the sample never consumed it. The interviewees were better inclined towards caffeine-containing soft drinks, with 64.7% drinking them sometimes, and less than 10% drinking them every day. A quarter of the sample never consumed this type of soft drink. Energy drinks were the least-consumed caffeine-containing product. Over 81% of the interviewees never consumed them, and only 0.4% consumed caffeine-containing energy drinks regularly.

Taste was the most relevant motive that drives people towards the consumption of caffeine-containing products. Consumers gave importance to mental energy provision, habit, and physical energy provision. The least important motives of the consumption of caffeine were socialization, symptom management, and mood enhancement (Table 4). The current research results support the proposition that caffeine consumption is strongly linked to its ability to supply physical and mental energy.

### 5.2. Consumers’ Intention to Consume Caffeine-Containing Products to Enhance Sport Performance

The results supported the proposition that consumers were fairly interested in purchasing caffeine-containing products to enhance sport performance (a mean value of 4.22 with a standard deviation of 1.53). Moreover, the consumers presented a negative attitude toward the consumption of caffeine-containing products to enhance sports performance (a mean value of 3.26 with a standard deviation of 1.53), a positive perception of their control over the consumption of caffeine-containing products to enhance sports performance (a mean value of 4.87 with a standard deviation of 1.36), a fairly-negative perception of the subjective norms regarding the consumption of caffeine-containing products to enhance sports performance (a mean value of 3.65 with a standard deviation of 1.24), and a positive consideration of the utilitarian values related to the use of caffeine-containing products to enhance sports performance (a mean value of 4.92 with a standard deviation of 1.23) (Table 5). Cronbach’s alpha values were calculated in order to assess the reliability and the internal consistency of the constructs (Table 6). Cronbach’s alpha values equal and higher than 0.7 were considered to be acceptable. Skewness and kurtosis tests were carried out in order to test the scores of normality. The values of skewness and kurtosis were within the acceptable ranges, with a sign of normality of the variables (skewness between −0.350 and −0.870, and kurtosis between −0.465 and 1.826) (Table 6). The possibility of issues surrounding non-normal distribution appeared to be insignificant.

These findings support the proposition that consumers tended to be positively inclined, and believed they were able to adequately manage the consumption of caffeine-containing products for sport performance enhancement. However, they are cautious, and do not fully rely on this type of product to enhance sport performance. They wondered whether the consumption of this type of product would be socially acceptable by friends and workgroups. They may tend to adopt a behavior conforming with social roles and norms. Understanding the perception of subjective norms is a powerful way of predicting how people will behave.

Next, we used a multivariate linear regression model in order to find possible existing correlations between the dependent variable of the model of this study (INT) and the independent construct variables in the application of the extended TPB model (ATT, SN, PBC and UD). A multi-collinearity analysis confirmed weak correlations among the independent variables. The correlation matrix of independent variables shows VIF values (Variance Inflation Factor) of around 1.4–1.5, and a tolerance of around 0.7 (Table 7). These values indicate that the level of correlation is low and, therefore, all of the independent variables are acceptable [50,51].

According to the results, the independent variables that were statistically significant at *p* < 0.05 were attitude (ATT), utilitarian drivers (UD), and subjective norms (SN). Each of them presented a positive correlation with the dependent variable, behavioral intention (INT). Subjective norms were the variable that has the highest impact on the behavioral intention (0.292), followed by utilitarian drivers (0.275) and attitude (0.163). Perceived behavioral control (PBC) was not statistically significant at *p* < 0.05. The goodness of fit of the statistical model was confirmed by the calculation of the R^2^ test and the F-test (Table 8). The values support the supposition that the independent variables fairly explain (R^2^ equal to 35.5%) the dependent variable’s variance. The limited difference between the R^2^ and the adjusted R^2^ value (34.6%) is satisfactory. The goodness of fit of the model is also confirmed by the F-test, which has an adequate *p* value, as it tends to 0.

The results supported the supposition that the intention to purchase caffeine-containing product to enhance sport performance was influenced by multiple factors, including the social context and norms that the consumers may be exposed to, the attitude that they themselves have toward this type of product, and the accessibility and ease of purchase of the product for the consumer.

### 5.3. Market Strategies for Caffeine-Containing Products to Enhance Sport Performance

The respondents showed a high level of interest for the specific effects of caffeine on sports performance. The consumers were generally rather positively inclined towards the effects that caffeine has on sport performance, with values ranging between 4.68 and 5.07 (Table 9). The most interesting effect for consumers was the improvement of neuromuscular coordination.

Among the possible product formats for the consumption of caffeine to enhance sports performance, traditional food and drinks containing caffeine (coffee, tea, chocolate and soft drinks), including energy drinks, were preferred by almost the half of the sample (47.4%). Furthermore, energy bars (46.5%) and ready-to-drink products (32.4%) were appreciated by the consumers. The consumers had limited interest in more innovative product formats, such as chewing-gum, pills, gels and soluble powders (Table 10). More traditional formats and snacking, such as energy bars, are the most interesting types of products for caffeine-containing produce.

About 70% of the interviewees stated a preference to buy caffeine-containing products to enhance sports performance at supermarkets. The second most appreciated purchasing outlets were pharmacies (33.2%), followed by the online market (25.7%), specialized shops (21.6%), sports supermarkets (17.1%), and finally gyms (10% of the sample) (Table 11). The preference of consumers towards supermarkets underlines the importance of the easy accessibility of the product. The results supported the supposition that the reliability of market channels, such as pharmacies, may improve the trustworthiness of caffeine-containing products to enhance sport performance, and possibly overcome consumers’ uncertainty on its safety and effectiveness. Finally, the research results supported the supposition that consumers would trust dieticians and personal trainers as advisors for the gathering of information and purchase advice about caffeine-containing products to enhance sports performance (respectively, for 61.5% and 59.3%) (Table 11). These results confirm consumers’ need to be supported in understanding how to adequately consume caffeine-containing products for sport performance.

## 6. Discussion

The results confirm that the most-consumed source of caffeine is by far coffee, followed by tea. Chocolate and soft drinks are consumed less frequently, and consumers do not drink energy drinks as a source of caffeine. These habits of caffeine-containing product consumption are in line with those found in previous studies [7,36,38]. As to the motives connected with the consumption of products containing caffeine, the most relevant were taste, mental energy supply, habit, and physical energy supply. These findings confirm the results achieved by Agòston [11]. His study supported that taste, habit and alertness were the first three main motives of caffeine consumption.

The primary purpose of the current research was to explore consumers’ intentions to consume caffeine-containing products to enhance sport performance. Thus, the research applied the TPB [40], which is widely adopted in consumer behavior research studies, including those on caffeine [52]. The model was extended in order to gain a more comprehensive understanding of consumers’ behavior towards caffeine as a stimulant to enhance sport performance.

The results support a rather positive intention to consume caffeine-containing products to enhance sport performance. Consumers showed a high self-confidence in their ability to control the consumption of caffeine-containing products, and a positive consideration of the utilitarian motives of caffeine consumption to enhance sport performance. However, the interviewees showed a cautious attitude, and a negative perception of the subjective norms related to the consumption of caffeine to enhance sport performance. Consumers are positively inclined, but hesitant. The results support the supposition that even younger people, as in the present study, may have to be reassured on the effects of caffeine-containing products to enhance sports performance.

The data elaboration tested the applied TPB model to predict the behavioral intention to consume caffeine to enhance sport performance. Subjective norms were a key determinant of the intention, suggesting that a positive or a negative perception of the subjective norms regarding the consumption of caffeine to enhance sports performance influences the intention to perform the analyzed behavior. The perceived subjective norms are negative, suggesting that there is the perception that people would not approve of such behavior. However, an increase in the subjective norms would lead to the highest likely change in the intention. Utilitarian Drivers were the second strongest predictor. Marketing levers—such as the ease of consumption, availability, affordability, and good taste—may contribute to the adoption of a positively-inclined behavior and the consumption of caffeine-containing products to enhance sports performance. Similar results were obtained in past studies [43]. The research adopted the TPB model in order to explore female athletes’ consumption of dietary supplements for sport performance, possibly also containing caffeine. Subjective norms emerged as the strongest predictor of the intention, which is consistent with the current research results [43].

The result of the consumers’ positive intention to consume caffeine-containing products to enhance sports performance confirms the consumers perception that caffeine can help achieve better performance, as highlighted by recent studies [7,44]. Consistently, ‘alertness’ is one of the consumers’ main drivers of caffeine consumption [11]. Caffeine-containing products, together with other sports nutrition products, can be considered part of a mainstreaming market phenomenon [28]. Sports nutrition products sales are expanding, with significant growth in more recent years [29].

Consumers have a great interest in the specific physiological effects of caffeine on sports performance, as identified by relevant scientific research [16,48]. The most appreciated effect was the improvement of the neuromuscular coordination. Frequently-consumed products containing caffeine—such as coffee, tea, chocolate, soft drinks, energy drinks, and snacking products, such as energy bars—were the favorite product formats, followed by ready-to-drink products. More innovative products—such as chewing-gum, gels, pills, and soluble powders—would not encounter consumers’ appreciation. Consumers associate caffeine with traditional food and beverage products, such as coffee, tea, and traditional beverages. Consumers struggle to consider caffeine a compound that is accessible in unusual food sources. It is worth noting that energy bars can be a promising food carrier of caffeine.

The favorite purchasing channel of caffeine-containing products to enhance sports performance is the supermarket. This finding confirms Bradley’s study’s results, which were promoted by the European Commission [31]. The present research highlights consumers’ appreciation of pharmacies as shopping outlets of caffeine-containing products to enhance sport performance, whereas the past study pointed out consumers’ interest in sport supermarkets and specialized shops [31].

Finally, the research results support the supposition that consumers would prefer to get information and purchase advice about caffeine-containing products to enhance sports performance from dieticians and personal trainers, rather than the web and friends. These findings show that consumers need to be reassured about the safety of the caffeine-containing products, and to receive reliable instructions on the adequate caffeine dosage to enhance sports performance.

## 7. Conclusions

The goal of this study was to analyze a specific consumer behavior: the consumption of food products containing caffeine for the purpose of enhancing sports performance. The findings of the current research provide a preliminary set of information that the food and sport nutrition sector companies may be interested in, in order to develop caffeine-containing products to enhance sports performance, and to fully operate in the ongoing sports nutrition market expansion. Caffeine can be found in products targeting people who practice physical activity, and those who want to enhance their performance. However, caffeine is only one of various other ingredients of performance-boosting food products. The caffeine-containing products that are marketed to enhance sports performance are still rare. Thus, there is room for companies’ expansion into this market. In particular, the current research supports the supposition that consumers should be educated on caffeine’s beneficial effects in sports, as the vast majority of consumers are not aware of this specific effect.

### 7.1. Managerial Implications

Currently, caffeine-containing food products to enhance sport performance are the prerogative of the sport food supplement industry. Coffee companies’ marketing strategies are mostly focused on taste, flavor, aroma, and consumption experience, and more recently on environmental and coffee-chain sustainability [7]. Frequently, coffee brands are sponsors for sport teams and events, including soccer, cycling, tennis, and basketball. The worlds of the coffee industry and the sport food supplement industry may get closer. Coffee roasters may fully exploit consumers’ perception that caffeine contributes to alertness and performance, and that coffee naturally contains caffeine, and their presence in sport initiatives to conceptualize, develop and expand their portfolio with a new product brand dedicated to coffee and sport performance.

Companies should take into account that an increasing number of people are adopting an active and healthy lifestyle. This leads to a rise of sport and endurance activities, and potentially also to a higher demand for products targeting physical performance enhancement. Caffeine is already promoted by the food industry, and used by consumers, for its natural low calorie content. Caffeine-containing supplements are positively perceived, and can be marketed to health and fitness-oriented consumers. A similar favorable perception may be fully operationalized for caffeine’s positive physical effects and sport performance.

Finally, in order to provide the necessary consumer safeguards in terms of food safety, food composition, consumer information, and legal certainty, the EU established that sports nutrition products are subject to the same legal requirements, and to the same level of harmonization, as other food falling under the horizontal rules of food law [53].

### 7.2. Limitations and Future Research

The current research may have some limitations. First, the results come from a convenience sample. Future studies may aim for samples with statistical representativeness, and may compare the perceptions of consumers living in different countries. Second, the research has an exploratory objective, providing preliminary results of an innovative food market phenomenon. Further research may aim for a better profiling of the consumer target. There is a need to better explore the tangible and intangible features of caffeine-containing food products for sport performance that consumers appreciate. Finally, there is a need to better understand what communication instruments and content should be conveyed to consumers in order to ensure appropriate caffeine consumption, with a specific focus on sport performance.

## Figures and Tables

**Table 1:** Literature references for the questionnaire items.

Questionnaire Item	Literature References
Section 1—Caffeine Consumption Habits and Physical Activity	
Types of caffeine-containing products consumption	[16]
Caffeine consumption and purchasing motives	[11]
Frequency of the physical activity	Self-developed
Types of sports	[16]
Section 2—Caffeine for sports performance: an application of the TPB	
Opinion on caffeine	Self-developed
EFSA opinion about caffeine for sports performance	[15]
Behavioral Intention items	[43]
Utilitarian drivers	Self-developed
Perceived Behavioral Control items	[43,47]
Subjective Norms items	[42,47]
Section 3—Marketing information about the product	
Caffeine effects on sport performance	[16,48]
Product formats	[31,32]
Favorite retail channel	[31]
Favorite purchasing advisors	[49]
Section 4—Socio-economic data	
Age	Self-developed
Gender	Self-developed
Level of education	Self-developed

Note: TPB (theory of planned behavior).

**Table 2:** Sample characteristics.

Characteristics	Types	%
Gender	Men	49.4
	Women	50.2
	Other	0.4
	Total	100
Age	Mean ± s.d. (years of age)	28.8 ± 10.9
Level of Education	Primary school	0
	Middle-school	4.2
	High-school	41.9
	Bachelor-Master degree	44.9
	PhD	9.1
	Total	100
Frequency of Physical Activity	Never	4.3
	Less than once/week	15.0
	Once a week	17.0
	More than once/week	49.8
	Every day	13.8
	Total	100

**Table 3:** Frequency of consumption of caffeine-containing traditional food and drinks.

	Coffee	Tea	Chocolate	Soft Drinks	Energy Drinks
Never	10.5	26	25.8	26.3	81.4
Less once/day	14.7	54.3	56.8	64.7	17.8
1/day	15.0	16.2	12.5	6.4	0.4
2/day	30.1	1.5	4.2	1.5	0.4
3/day	19.9	1.1	0.4	0.4	0
4/day	6.4	0.4	0	0	0
5/day or more	3.4	0.4	0.4	0.8	0
Total	100	100	100	100	100

**Table 4:** Motives of caffeine consumption.

	Mean
I like the taste of products with caffeine	4.82
Caffeine supplies me with mental energy	4.42
I have the habit of consuming caffeine	4.15
Caffeine supplies me with physical energy	4.00
Caffeine puts me in a better mood	3.46
Caffeine reduces some symptoms	2.72
Caffeine helps me socialize	2.57

Note: the level of relevance is based on a 1­–7 Likert scale, from ‘completely not relevant’ (1) to ‘completely relevant’ (7).

**Table 5:** TPB constructs and items.

Constructs and Items	Mean Value	Standard Deviation
Behavioral Intention (INT)	4.22	1.53
I might be interested in buying caffeine-containing products to enhance my sports performance	3.82	1.51
I might be interested in consuming caffeine-containing products to enhance my sports performance	3.85	1.52
I might be interested in consuming caffeine-containing products to enhance my sports performance if I am sure they work	4.31	1.57
I might be interested in consuming caffeine-containing products to enhance my sports performance if I know they are safe and tested	4.83	1.53
I might be interested in consuming caffeine-containing products to enhance my sports performance if I can afford them	4.28	1.52
Attitude (ATT)	3.26	1.3
Consuming caffeine-containing products is a good way to enhance sports performance for people who practice physical activity	4.02	1.29
People practicing physical activity need caffeine-containing products to enhance sports performance	3.30	1.32
People practicing physical activity need caffeine-containing products for health and wellbeing reasons	3.04	1.21
Caffeine-containing products work because they are consumed by people practicing physical activity	2.68	1.38
Perceived Behavioral Control (PBC)	4.87	1.36
Consuming caffeine-containing products is completely under my control	5.51	1.53
I am able to consume caffeine-containing products if I want to	5.81	1.23
Consuming caffeine-containing products makes me feel like I am taking care of my physical performance	3.34	1.40
Consuming caffeine-containing products is easy for me	4.81	1.28
I can consume caffeine-containing products from now on if I want to	4.88	1.37
Subjective Norms (SN)	3.65	1.24
My personal trainer would support the use of caffeine-containing products to enhance sports performance	3.79	1.14
My family would support the use of caffeine-containing products to enhance sports performance	3.39	1.28
My teammates would support the use of caffeine-containing products to enhance sports performance	4	1.32
My doctor would support the use of caffeine-containing products to enhance sports performance	3.36	1.27
Many people who are important to me would support the use of caffeine-containing products to enhance sports performance	3.71	1.2
Utilitarian Drivers (UD)	4.92	1.23
Caffeine-containing products should be easily available.	4.59	1.31
Caffeine-containing products should be easy to take.	4.84	1.17
Caffeine-containing products should taste good.	5.28	1.33
Caffeine-containing products should be affordable.	5	1.12

**Note:** Each scale utilized a 7-point Likert type scale ranging from completely disagree (1) to completely agree (7). The constructs were considered to be negative at values below 4 (ATT, SN), and constructs were considered to be positive at values equal to or above 4 (INT, PBC and UD).

**Table 6:** Cronbach’s alpha, Skewness and Kurtosis of the TPB constructs.

Construct	N of Items	Cronbach’s Alpha	Skewness	SE	Kurtosis	SE
Behavioral Intention	5	0.92	−0.721	0.149	−0.177	0.297
Attitude	4	0.79	−0.350	0.149	−0.465	0.297
Perceived Behavioral Control	5	0.71	−0.870	0.150	1.826	0.299
Subjective Norms	5	0.89	−0.679	0.150	0.411	0.298
Utilitarian Drivers	4	0.81	−0.510	0.150	1.250	0.298

**Table 7:** Multivariate linear regression of the model constructs.

	Unstandardized Coefficient		Standardized Coefficients	t	Sig.	95.0% Confidence Interval for B		Multi-Collinearity Statistics	
	B	Std.Error	Beta			Lower Bound	Upper Bound	Tolerance	VIF
(Constant)	−0.48	0.409		−0.116	0.908	−0.853	0.758		
ATT	0.216	0.080	0.163	2.686	0.008 *	0.058	0.374	0.666	1.502
SN	0.379	0.077	0.292	4.914	0.000 *	0.227	0.531	0.695	1.439
PCB	0.067	0.087	0.046	0.775	0.439	−0.103	0.238	0.686	1.458
UD	0.375	0.081	0.275	4.663	0.000 *	0.217	0.534	0.708	1.412

Note: Dependent Variable: intention to purchase caffeine-containing products to enhance sport performance; *statistically significant at *p* < 0.05. VIF: variable inflation factors; ATT: attitude; SN: subjective norms; PCB: perceived behavioral control; UD: utilitarian drivers.

**Table 8:** R2 test and F-Test.

Model	R	R Square		Adjusted R Square	Std. Error of the Estimate
1	0.596	0.355		0.346	1.0766
Model	Sum of Squares	df	Mean Square	F	Sig.
1 Regression	167.466	4	41.866 1.159	36.121	0.000
Residual	303.674	262			
Total	471.140	266			

Note: The assessment of the model’s fit was positive, as supported by the value of the standard error of the estimate (1.0766) being equal to the square root of the residual (1.159).

**Table 9:** Level of interest in the specific effects of caffeine on sports performance.

Specific Effects	Mean	Standard Deviation
Better neuromuscular coordination	5.07	1.52
Reflexes improvement	4.87	1.57
Increase of the accuracy	4.85	1.53
Increase of the speed	4.76	1.51
Fatigue reduction	4.71	1.63
Better control of the sports equipment	4.68	1.57

Note: The level of interest is based on a 7-point Likert scale, from absolutely not interested (1) to absolutely interested (7).

**Table 10:** Perception of caffeine-containing products’ formats for sports performance enhancement.

Product Formats	N° of Responses	% of Responses	% on the Sample
Energy bars	117	26.8	46.5
Soluble powders	21	4.8	7.8
Ready to drink products (energy-shots)	87	19.9	32.4
Pills	30	6.9	11.1
Chewing-gum	32	7.3	11.9
Common food and drinks	127	29.1	47.4
Gels	23	5.3	8.6
Total	437	100	

Note: Multiple choice questions with a yes/no answer for each item.

**Table 11:** Consumers’ favorite retail channels, purchasing advisors for the gathering of information, and purchase advise about caffeine-containing products for sport performance.

Retail Channels	N° of Responses	% of Responses	% of the Sample
Supermarkets	187	39.3	69.7
Sports supermarkets	46	9.7	17.1
Specialized shops	58	12.2	21.6
Pharmacies	89	18.7	33.2
Gyms	27	5.7	10
Online	69	14.5	25.7
Total	476	100	
Purchasing Advisors	N° of Responses	% of Responses	% of the Sample
Dietician	165	42.6	61.5
Personal trainer	159	41.1	59.3
Web	14	3.6	5.2
Friends	20	5.2	7.5
Other	29	7.5	10.8
Total	387	100	

Note: Multiple choice questions with a yes/no answer for each item.

## Data Availability

The data that support the findings of this study are available from the corresponding author upon request.
